# Comparison of MRCP and ERCP in the evaluation of common bile duct and pancreatic duct pathologies

**DOI:** 10.3389/fmedt.2023.946555

**Published:** 2023-07-12

**Authors:** Anand Kumar, Nihar Ranjan Mohanty, Madhusmita Mohanty, Sashibhusan Dash

**Affiliations:** ^1^ Department of Radiodiagnosis, Apollo Hospitals, Bhubaneswar, India; ^2^ Department of Radiodiagnosis, Kalinga Institute of Medical Sciences (KIMS), Bhubaneswar, India; ^3^ Department of Pathology, Kalinga Institute of Medical Sciences (KIMS), Bhubaneswar, India; ^4^ Department of Oncopathology, Acharya Harihar Postgraduate Institute of Cancer (AHPGIC), Cuttack, India

**Keywords:** magnetic resonance cholangiopancreatography, endoscopic retrograde cholangiopancreatography, biliary pancreatic region, gold standard, gastrointestinal disorders

## Abstract

**Background:**

Magnetic resonance cholangiopancreatography (MRCP) is a non-invasive imaging modality that has high diagnostic accuracy for a wide range of bile duct and pancreatic duct pathologies. Endoscopic retrograde cholangiopancreatography (ERCP) is still the gold standard for the exploration of the biliopancreatic region.

**Aim:**

The aim of the study was to compare the diagnostic accuracy of MRCP with that of ERCP in the diagnosis of bile duct and pancreatic duct pathologies.

**Material and methods:**

A total of 60 patients with common bile duct (CBD) and pancreatic duct pathologies detected on MRCP were subsequently evaluated by ERCP in this observational study. A comparison of MRCP findings with ERCP was made.

**Results:**

MRCP had a sensitivity, specificity, positive predictive value (PPV), negative predictive value (NPV), and accuracy of 88.1%, 94.4%, 97.3%, 72.7%, and 90%, respectively, in diagnosing choledocholithiasis in comparison to ERCP. For CBD dilation, the sensitivity was 90.91%, specificity was 93.75% and the PPV, NPV, and accuracy were 97.56%, 78.95%, and 91.67%, respectively, for MRCP. In CBD stricture, MRCP showed a sensitivity, specificity, PPV, NPV, and accuracy of 83.33%, 97.92%, 90.91%, 95.92%, and 95%, respectively. In pancreatic duct dilatation, the sensitivity, specificity, PPV, NPV, and accuracy were all 100%. Pancreatic duct stricture showed a sensitivity, specificity, PPV, NPV, and accuracy of 80%, 98%, 88.89%, 96.08%, and 95%, respectively. For the diagnosis of periampullary carcinoma, the sensitivity, specificity, PPV, NPV, and accuracy rate of MRCP were 80%, 98%, 88.89%, 96.08%, and 95%, respectively.

**Conclusion:**

No significant difference was found between MRCP and ERCP in diagnosing those six pathologies.

## Introduction

Accurate methods of detecting common bile duct (CBD) and pancreatic disease in patients are important to both surgeons and endoscopists for planning an effective interventional strategy; therefore, there is a need for less invasive, safe and highly sensitive diagnostic procedures.

Various invasive and non-invasive diagnostic techniques have been employed to achieve this aim. Non-invasive techniques, such as ultrasound and CT scanning (abdomen and pelvis) are widely used in the preliminary investigations of pancreaticobiliary disease. These techniques, though easily available and less expensive, have limitations in terms of sensitivity, such as the low sensitivity of ultrasonography for detecting common duct calculi, which means that the diagnosis of several common conditions, such as tumours, calculi, sclerosing cholangitis and chronic pancreatitis, may require invasive procedures (1).

Endoscopic retrograde cholangiopancreatography (ERCP) is often regarded as a definitive diagnostic test (2, 3). ERCP combines the use of endoscopy and fluoroscopy and has additional therapeutic advantages (3). Although currently ERCP is almost exclusively used for therapeutic purposes, it is still regarded as an important tool in the evaluation of biliary diseases, especially malignancy (4). As ERCP is invasive, the associated disadvantages are as follows: (1) it requires direct cannulation of the common bile duct or pancreatic duct; (2) it requires sedation; (3) it is more operator-dependent, requiring more experienced personnel; and (4) it uses ionising radiation. ERCP is also associated with a complication rate of 1%–7%, including haemorrhage, sepsis, pancreatitis, and bile leak. About one in four complications is severe. The overall complication rate appears relatively consistent across time (2–4).

In the evaluation of biliary and pancreatic duct anatomy and obstruction, magnetic resonance cholangiopancreatography (MRCP) is a non-invasive, radiation-free, non-operator-dependent, multiplanar, and safe alternative to diagnostic ERCP (5). MRCP was developed in 1991, and techniques have improved since then. MRCP makes use of heavily T2-weighted sequences, which significantly increase the signal of static or slow-moving fluid-filled structures, such as bile and pancreatic ducts, and that leads to increased duct to background contrast (6). The most recent software available includes fast, high spatial resolution MRCP sequences, such as a heavily T2-weighted turbo-spin-echo (TSE), single-shot rapid acquisition with relaxation enhancement (RARE), and half-Fourier single-shot TSE (HATSE), which provide clear projectional images that are similar to those provided by ERCP procedures (7).

According to recent studies, the diagnostic accuracy of MRCP is comparable with that of ERCP for the evaluation of extrahepatic bile duct and pancreatic duct abnormalities, such as choledocholithiasis, malignant obstruction of the bile and pancreatic ducts, congenital anomalies, chronic pancreatitis, benign strictures due to sclerosing cholangitis, demonstrating pancreatic pseudocyst, and in cases of biliary cystadenoma and cystadenocarcinoma (7, 8). The advantages of MRCP include the following: 3D imaging and image reformatting; it is non-invasive; patients tolerate it well; and it is an excellent diagnostic tool in situations where ERCP is difficult, hazardous, or impossible (7).

In our study, we put forward the hypothesis that MRCP can provide relevant information regarding CBD and pancreatic duct pathology. The aim of the study was to compare the diagnostic accuracy of MRCP with that of ERCP in common bile duct and pancreatic duct pathologies and to correlate both techniques.

## Materials and methods

The study was conducted in the Department of Radiodiagnosis, Apollo Hospital Bhubaneswar, the Department of Radiodiagnosis, Kalinga Institute of Medical Sciences, Bhubaneswar, and the Department of Gastroenterology and Hepatology, Apollo Hospital Bhubaneswar, over a period of 1 year, between December 2018 and November 2019. It was a prospective observational study in which patients with common bile duct or pancreatic duct pathologies detected in MRCP were evaluated by ERCP.

The inclusion criteria for our study included patients with CBD pathologies (choledocholithiasis, CBD stricture, CBD dilatation), pancreatic duct pathologies (pancreatic duct stricture, pancreatic duct dilatation), and periampullary carcinomas (those that arise from the head of the pancreas, the distal common bile duct, and within 2 cm of the major papilla in the duodenum) detected on MRCP who were subsequently evaluated by ERCP.

The exclusion criteria for our study were patients with absolute contraindications to the MRCP technique, such as patients with incompatible implants, patients with claustrophobia, patients with CBD, and pancreatic duct pathologies, in whom therapeutic interventions were not indicated, any case that developed complications during the procedure and all cases of failed cannulation.

CBD dilatation in adults is defined as a common bile duct measuring ≥8 mm at its widest part. A dilatation of 8 mm–12 mm is mild, 12 mm–16 mm moderate, 16 mm–20 mm severe, and >20 mm extremely severe.

CBD strictures are narrowing segments of the intrahepatic or extrahepatic biliary ductal system. The narrowing impedes the normal antegrade flow of bile, causing proximal dilatation.

A dilated main pancreatic duct was diagnosed when it measured >3 mm in the head and <2 mm in the body and tail regions of the pancreas.

This study protocol was approved by Institutional Ethics Committee Apollo Hospitals, Bhubaneswar (registration no. ECR/246/Inst/OR/2013/RR 2016 on 12 April 2019). The studies were conducted in MRI and ERCP units.

### MRCP and MRI techniques

In this study, an eight-channel 1.5-T MRI scanner (Signa HDxt; GE Healthcare) using torso phased-array coils was used for the MRCP techniques. Pre-procedural preparation included 6 h of fasting to promote gallbladder filling.

(1) Three phase gradient—echo localising images were obtained and used to plan the MRCP sequences.
(2) Axial slices were performed using single shot fast spin-echo (SSFSE) sequences:
  • Parameters: TE=90 ms,
  • Field of view (FOV): 28 cm–38 cm,
  • Slice thickness: 10 mm,
  • Spacing: 2 mm,
  • Frequency: 256 kHz,
  • Phase encoding FOV: 8 cm, and
  • Frequency of encoding direction: right to left.
(3) The following sequences were also obtained:
  • Ax T2 FRFSE RTr Fat SAT,
  • COR T2 SSFSE RTr ASSET,
  • 3D MRCP RTr ASSET (coronal section in plane with CBD and axial section in plane with pancreatic duct),
  • THIN COR 2D FIESTA FAT SAT,
  • AX LAVA-XVBH,
  • Thick slab MRCP,
  • SAG T2 SSFSE RTr ASSET, and
  • AX DW RTr b1000.

All the sequences were required during a single breath-hold. The entire examination was usually completed within 20 min. MRCP was performed before ERCP and the results were evaluated by senior consultant radiologists.

### ERCP technique

ERCP was performed using an Olympus CV150 duodenoscope, and fluoroscopic images were obtained using a Philips BV Libra system. Pre-procedural preparation included at least 12 h of fasting. Omnipaque™ (iohexol) contrast was used and the procedure was performed under anaesthesia using propofol. ERCP was performed by a well-trained and experienced endoscopist. Cholangiograms were obtained.

### Statistical analysis

Data were collected on different pancreato-biliary pathologies. The diagnostic test evaluation calculator MEDCALC software was used to calculate the outcome measures of sensitivity, specificity, positive predictive value (PPV), negative predictive value (NPV), and accuracy of MRCP with reference to ERCP as the gold standard in respect to different pancreato-biliary pathologies. A chi-square test was used to compare both groups (i.e., cholangiography and pancreatography) using IBM SPSS Statistics 24.0 software (SPSS South Asia Pvt. Ltd).

## Results

A total of 60 patients (46.7% men, 53.3% women; age range 23–79 years; mean age 54.35 ± 29.62 years) were included in the study. The majority of cases (46.7%) belonged to the 50–60-year age group, with nearly one-quarter belonging to the 65 years and older age group.

In this study, most patients presented with severe abdominal pain, obstructive jaundice, unexplained fever, anaemia, and weight loss. Six different pathologies, such as those of the common bile duct and pancreatic duct, emerged according to ERCP. The two most common pathologies were CBD dilatation (73.3%) and choledocholithiasis (70%). Other pathologies were CBD stricture (20%), pancreatic duct dilatation (18.3%), pancreatic duct stricture (16.7%), and periampullary neoplasms (16.7%).

### Choledocholithiasis

On MRCP, the location, size, and number of stones were in accordance with the ERCP examination. The stone size was <5 mm in 59% of cases, while stones >5 mm were observed in 41%. MRCP correctly diagnosed 37 of 42 patients with ERCP-proven CBD calculi and 17 of 18 patients without calculi. Of the 60 cases, 37 were true positives, one was a false positive, five were false negatives, and 17 were true negatives.

In our study, the bile duct diameter (mean = 8.6 mm) was measured in the setting of choledocholithiasis. Of the five false-negative MRCP cases, all had dilated bile ducts >10 mm on ERCP. The sensitivity was high (88.1%, 95% CI: 74.37–96.02), as was the specificity (94.44%, 95% CI: 72.71–99.86) with reference to ERCP. The PPV, NPV, and accuracy were 97.37%, 77.27%, and 90%, respectively.

### CBD dilatation

MRCP correctly diagnosed 40 of 44 ERCP-proven cases and excluded 15 of 16 unaffected patients. Out of 60 cases, 40 were true positives, one was a false positive, four were false negatives, and 15 were true negatives. The sensitivity was very high (90.91%, 95% CI: 78.33–97.47), as was the specificity (93.75%, 95% CI: 69.77–99.84) with reference to ERCP. The PPV, NPV, and accuracy were 97.56%, 78.95%, and 91.67%, respectively.

### CBD stricture

**Figure 1:**
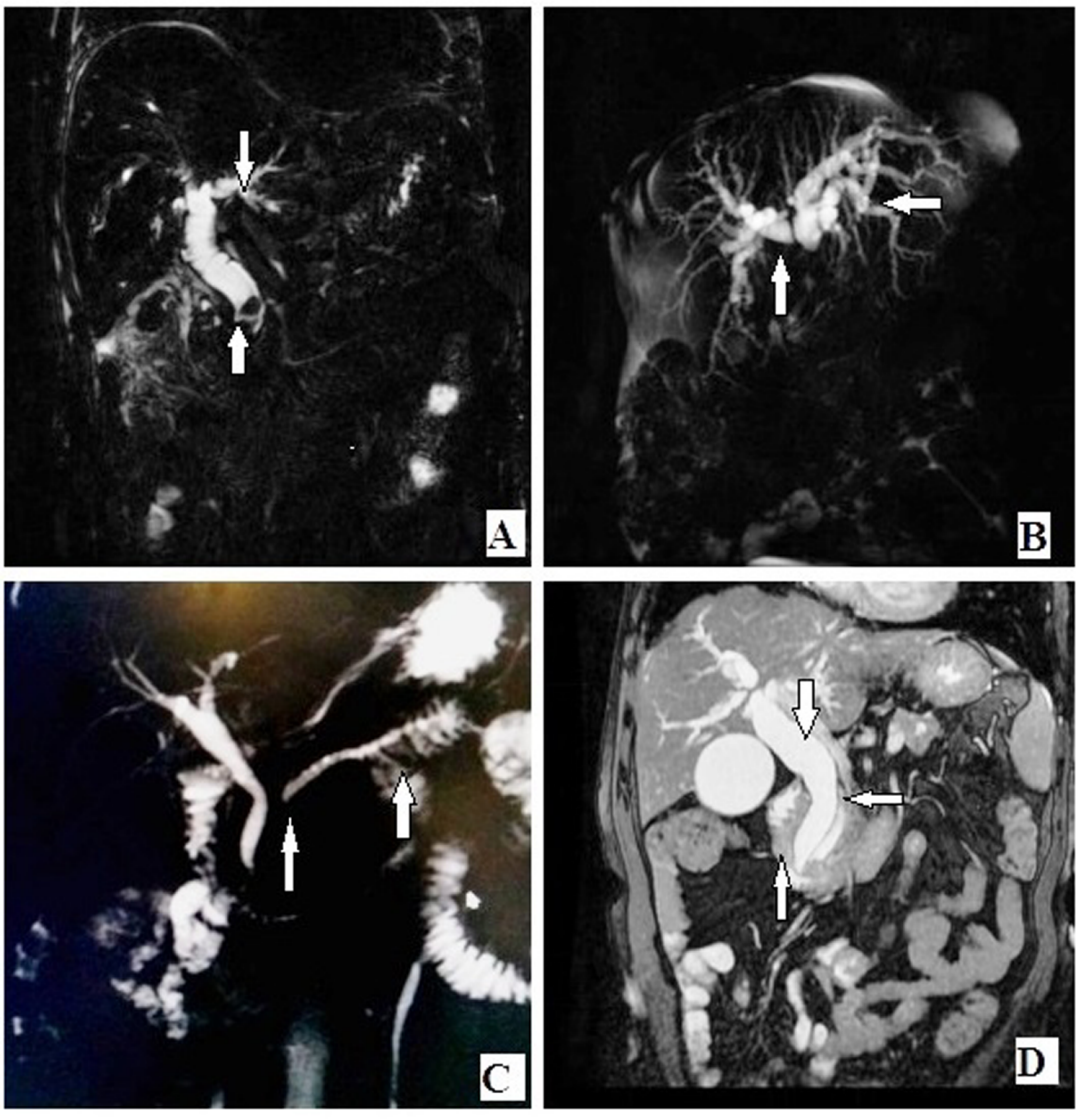
(**A**) Coronal MIP reformat showing CBD calculus at distal end with proximal obstructive bile duct dilatation. (**B**) Coronal MIP reformat showing hilar mass lesion with proximal bile duct dilatation and non-confluence of right and left hepatic ducts. (**C**) Coronal MIP reformat showing stricture of MPD with upstream dilatation in a case of chronic pancreatitis. (**D**) Coronal T2-weighted fat-saturated image showing neoplastic mass of periampullary region with stricture of distal CBD and distal MPD and dilatation of proximal pancreatic duct and proximal CBD and IHBR. CBD, common bile duct; MIP, maximum intensity projection; MPD, Main pancreatic duct; IHBR, Intrahepatic biliary radicals.

ERCP-proven CBD strictures were correctly diagnosed by MRCP in 10 out of 12 ERCP-proven cases, and MRCP correctly excluded stricture in 47 cases. The true-positive, true-negative, false-positive, and false-negative results were 10, 47, 1, and 2, respectively. The sensitivity and specificity were high at 83.33% (95% CI: 51.59–97.91) and 97.92% (95% CI: 88.93–99.95), respectively, with reference to ERCP. The PPV, NPV and accuracy were 90.91%, 95.92%, and 95.9%, respectively (Figure 1A,B).

### Dilatation of the pancreatic duct

MRCP correctly diagnosed 11 cases, the same number of cases as diagnosed by ERCP. No false-positive or false-negative cases were seen. Both techniques correctly excluded 49 unaffected cases. The sensitivity, specificity, PPV, NPV, and accuracy were 100% (95% CI: 71.51–100), 100% (95% CI: 92.75–100), and 100%, respectively.

### Stricture of the pancreatic duct

Of the 10 pancreatic duct stricture cases diagnosed by ERCP, MRCP correctly diagnosed eight cases, and of 50 unaffected patients, MRCP excluded 49 cases. The numbers of true-positive, true-negative, false-positive, and false-negative cases reported by MRCP were 8, 49, 1, and 2, respectively. The sensitivity was high (80%, 95% CI: 44.39–97.48), as was the specificity (98%, 95% CI: 89.35–99.95) with reference to ERCP. The PPV, NPV, and accuracy were 88.89%, 96.08%, and 95%, respectively.

### Periampullary carcinoma

MRCP correctly diagnosed 9 out of 10 cases diagnosed by ERCP. MRCP correctly excluded 50 cases out of 50 unaffected cases. The numbers of true-positive, false-positive, true-negative, and false-negative cases were 9, 0, 50, and 1, respectively. With reference to ERCP, the sensitivity was high at 90% (95% CI: 55.50–99.75) and the specificity was high at 100% (95% CI: 92.89–100.00). The PPV, NPV, and accuracy were 100%, 98.04%, and 98.33%, respectively. The correlation of MRCP and ERCP in the evaluation of the six different pathologies is shown in Table 1.

**Table 1:** Correlation of MRCP and ERCP with reference to different pathology.

Pathology	MRCP		ERCP		*p*-Value
	Present	Absent	Present	Absent	
CDL	38	22	42	18	>0.05
CBDD	41	19	44	16	
CBDS	11	49	12	48	
PDD	11	49	11	49	
PDS	9	51	10	50	
PAC	9	51	10	50	

CDL, choledocholithiasis; CBDD, common bile duct (CBD) dilatation; CBDS, CBD stricture; ERCP, endoscopic retrograde cholangiopancreatography; MRCP, magnetic resonance cholangiopancreatography; PAC, periampullary carcinoma; PDD, pancreatic duct dilatation; PDS, pancreatic duct stricture.

**Figure 2:**
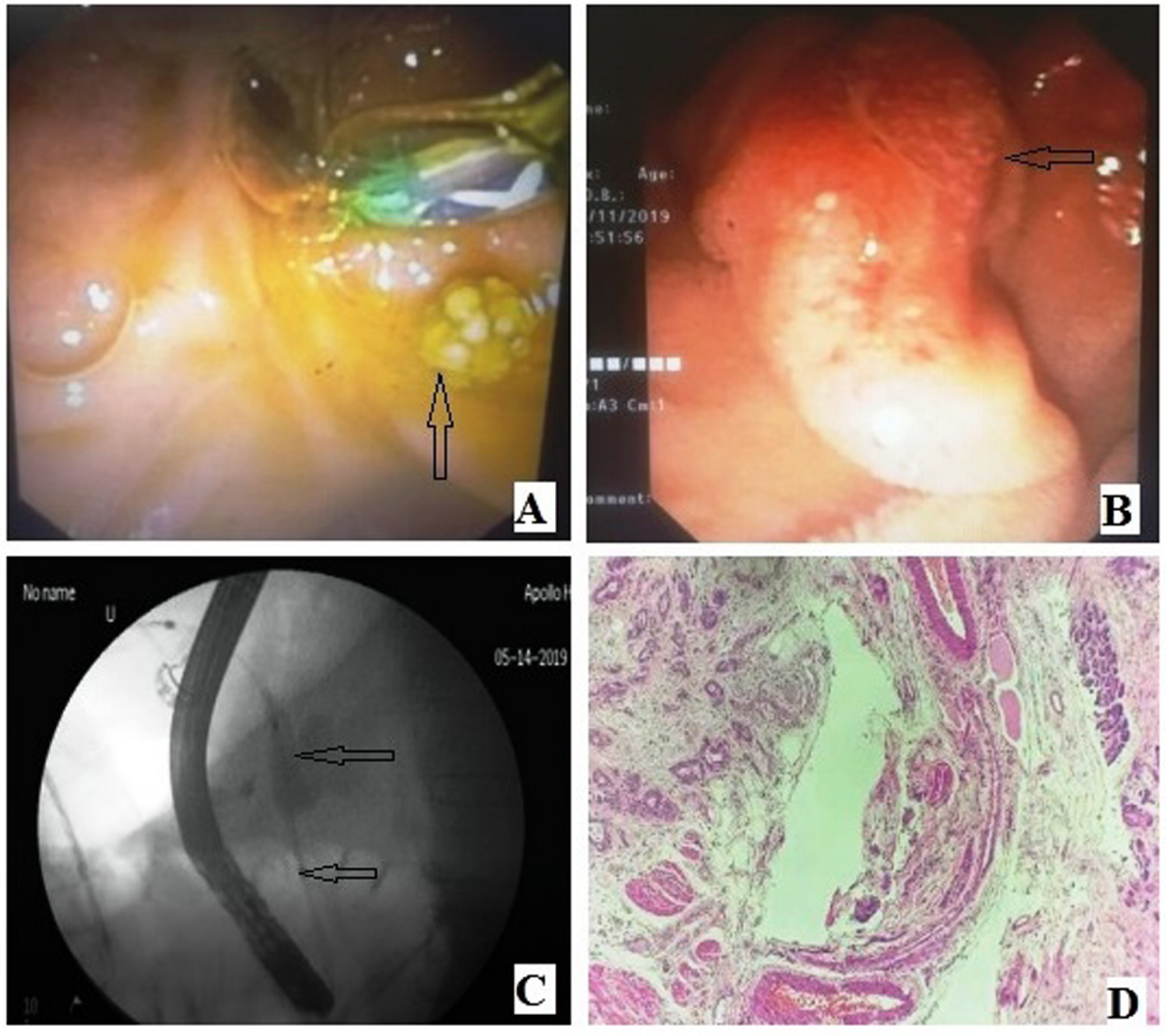
(**A**) ERCP duodenoscopic image showing CBD calculus. (**B**) ERCP duodenoscopic image: Periampullary neoplasm. (**C**) ERCP fluoroscopic image: CBD stricture with dilatation of proximal biliary tree. (**D**) Haematoxylin and eosin (40×): Periampullary adenocarcinoma at left-hand side and normal pancreatic acini at right-hand side. CBD, common bile duct; ERCP, endoscopic retrograde cholangiopancreatography.

Choledocholithiasis was found in 63% of cases of MRCP and 70% of cases of ERCP. MRCP and ERCP detected CBD strictures in 18% and 20% of cases, respectively. Both techniques detected pancreatic duct dilatation in 18% of cases each and pancreatic duct stricture in 15% and 16% of cases, respectively. Periampullary carcinoma was diagnosed in 15% and 16% of MRCP cases, respectively. There was no significant difference between MRCP and ERCP in the evaluation of these pathologies (Figures 1 and 2).

## Discussion

In our study, the elderly age group (>50 years) is commonly affected by these pathologies (*p* = 0.001), which is consistent with the study conducted by Kimura et al. (8). The number of affected women is slightly higher than that of affected men in the present study, which correlates with the study conducted by Ko et al. (9). According to O’Connor et al., choledocholithiasis and CBD dilatation are some of the most common biliary tract pathologies (1), which matches the findings of our study.

In this study, MRCP correctly diagnosed 37 of 42 patients with ERCP-proven CBD calculi and 17 of 18 patients without calculi. The location, size, and number of stones were consistent with the ERCP examination. According to Griffin et al. (10), compared with ERCP, MRCP has a sensitivity, specificity, PPV, and NPV of 86%, 93%, 87%, and 82%, respectively, in diagnosing choledocholithiasis. According to Vitellas et al. (6), MRCP is comparable to ERCP in diagnosing choledocholithiasis, with sensitivities and specificities in the range of 81%–100% and 85%–100%, respectively. In another study, the sensitivity of MRCP and ERCP for identifying choledocholithiasis was 80% and 90%, respectively. The overall agreement between MRCP and ERCP was 90.6% (11).

The results of our study show that the diagnostic accuracy of MRCP in detecting choledocholithiasis was 90% and comparable to the results of previous studies (6, 10–12). A false-negative diagnosis had occurred, as multiple small (2 mm) intrahepatic duct stones were missed on the MRCP. Stones were probably missed because of the lack of contrast between the stones and surrounding liver, with no high signal bile outlining the stones.

It is known that the sensitivity of MRCP for detecting choledocholithiasis decreases with bile duct dilatation (72% for bile duct diameter >10 mm vs. 88.9% for diameter ≤10 mm) (11, 13). In our study, five false-negative cases on MRCP had dilated bile ducts >10 mm on ERCP examination.

According to Chan et al., in diagnosing CBD dilatation, MRCP showed a sensitivity of 95%, specificity of 85%, PPV of 82%, and NPV of 96% compared to ERCP (14). Hintze et al. found the sensitivity and PPV of MRCP in detecting bile duct dilatation to be 83% and 91%, respectively (15). Our results show high parameters and are in accordance with previous studies (4, 12).

The CBD stricture was seen as a narrowing of the CBD with upstream biliary dilatation. CBD stricture includes a few cases of benign causes, such as infections and pancreatitis, and malignant causes, such as pancreatic neoplasm, periampullary tumour, and cholangiocarcinoma. According to Hintze et al., considering ERCP as the gold standard, MRCP showed a sensitivity and PPV of 85% and 100%, respectively, in diagnosing bile duct stricture (15). Lomas et al. found MRCP to be highly accurate in diagnosing biliary stricture, with a sensitivity of 100% and specificity of 98% (16). The results of our study show the sensitivity and specificity to be 83.33% and 97.92%, respectively, with reference to ERCP. The PPV, NPV, and accuracy were 90.91%, 95.92%, and 95.9%, respectively.

According to Coakley et al., MRCP has a sensitivity of approximately 87%–100% for pancreatic duct dilatation (17). Soto et al. found MRCP to have a sensitivity of 100% and 87% (observers 1 and 2, respectively) for diagnosing pancreatic duct dilatation (18). Takehara et al. also found an agreement of 83%–92% for diagnosing pancreatic duct dilatation by MRCP and ERCP (19). In our study, the sensitivity, specificity, PPV, NPV, and accuracy of MRCP in detecting pancreatic duct dilatation were in accordance with the results of the above-mentioned studies.

According to Hintze et al. (15), MRCP has a sensitivity and PPV of 76% and 87%, respectively, in the recognition of pancreatic duct stricture compared to ERCP. According to Hurter et al. (20), in diagnosing pancreatic duct stricture, MRCP has a sensitivity of 100%, PPV of 94.1% and NPV of 100%. In this study, the sensitivity was 80% (95% CI: 44.39–97.48) and the specificity was 98% (95% CI: 89.35–99.95) with reference to ERCP. The PPV, NPV, and accuracy were 88.89%, 96.08%, and 95%, respectively.

According to Sugita et al. (21), the sensitivity, specificity, accuracy, PPV, and NPV of high-resolution MRI for the evaluation of periampullary carcinoma were 88%, 100%, 96%, 100%, and 94%, respectively. They observed that MRCP can accurately detect the location, extension, and origin of periampullary carcinoma and is beneficial in the preoperative staging of tumours.

According to Pamos et al. (22), the sensitivity and specificity of MRCP compared to ERCP in diagnosing periampullary carcinoma were 100% and 83%, respectively. In our study, the results for MRCP in diagnosing periampullary carcinoma were high (sensitivity = 90%, specificity = 100%) and comparable to the results of the above-mentioned studies. MRCP exhibited a high level of diagnostic precision for obstructive jaundice.

According to the most recent studies, MRCP has the potential to replace diagnostic ERCP in a wide variety of bile duct anomalies (bile stones, benign and/or malignant strictures, CBD), which would be a significant advancement in the field as this could reduce the frequency of invasive procedures undergone by the patient. This would curtail the occurrence rate of potential complications associated with ERCP (4, 12).

The present study has some limitations. A small sample size is an important limitation. This is due to the limited duration of the study and the difficulty in finding cases that underwent both MRCP and ERCP, as only those patients with therapeutic interventions indicated were subjected to ERCP examinations. There are various CBD and pancreatic duct pathologies. However, in our study, because of the above-mentioned limitations, we could evaluate the diagnostic accuracy of only six pathologies.

## Conclusion

MRCP, with reference to ERCP, has high sensitivity, specificity, PPV, NPV, and accuracy, with no significant difference in diagnosing those six pathologies. In the case of stricture of the CBD and pancreatic duct, MRCP can accurately reveal the location and extent of stricture. MRCP offers the additional advantage of cross-sectional imaging in cases of periampullary neoplasm and can accurately evaluate tumour invasion into surrounding tissue.

## Data Availability

The original contributions presented in the study are included in the article, further inquiries can be directed to the corresponding author.
